# From statistics to stories: understanding the complex landscape of missed medical appointments. A mixed-methods pilot study

**DOI:** 10.3399/BJGPO.2024.0007

**Published:** 2024-09-18

**Authors:** Lea Charton, Francis Gatier, Chloe Delacour, Camille Lépine

**Affiliations:** 1 General Medicine Department, Faculty of Medicine, University of Strasbourg, Strasbourg, France; 2 Maison de Santé Pluriprofessionnelle Universitaire du Neuhof, Strasbourg, France

**Keywords:** inequalities, community care, health inequities, general practice, pilot projects

## Abstract

**Background:**

Research suggests that in both France and the UK, between 5% and 10% of appointments with GPs are unattended. A comprehensive Irish study linked missed appointments with an increased short-term risk of mortality, prompting further investigation into the reasons behind absenteeism.

**Aim:**

To delve into the underlying causes of missed appointments, within the context of an urban health centre.

**Design & setting:**

Using a mixed-method approach, this study combines qualitative telephone interviews with quantitative analysis of medical records. The study focuses on patients who failed to attend appointments at an urban health centre in France over a 15-day period.

**Method:**

The interview guide collected data on circumstances leading to missed appointments and explored patients' social determinants of health. Additionally, quantitative data, including patients’ socioeconomic backgrounds, were extracted from medical records.

**Results:**

Among 53 missed appointments (4.9% of all scheduled), 22 patients were interviewed. State health coverage (SHC) beneficiaries (68.2% of the sample) cited socioeconomic instability, including precarious work hours, social isolation, and multiple commitments, as reasons for non-attendance. For non-SHC beneficiaries, forgetting the appointment or failing to cancel it after self-resolution of the health issue was one of the main causes. Remarkably, 36.4% disclosed that they had experienced domestic violence. During the qualitative interview, patients were offered the opportunity to reschedule appointments, and a retrospective analysis by physicians found that over a quarter of the missed appointments were classified as 'capital appointments', meaning their absence could have significantly harmed the patient's health.

**Conclusion:**

The findings indicate that missed appointments can highlight social inequality, emphasising the need to align health care with patients' temporal realities. The identification of patients who have experienced violence and the use of missed appointments as triggers for follow-up calls seem to be promising strategies to enhance care and mitigate health inequalities.

## How this fits in

Before this study, it was understood that a significant number of medical appointments in France were not honoured, primarily by beneficiaries of state health coverage (SHC). This research provides valuable insights into the multifaceted reasons behind appointment non-attendance, which is often intertwined with socioeconomic factors. Moreover, it highlights a possible connection between missed appointments and patient vulnerability, potentially leading to short-term mortality risk. Clinicians can leverage the findings of this research to better grasp and address the underlying factors contributing to appointment non-attendance, ultimately enabling improved patient support, particularly for those grappling with social and economic challenges.

## Introduction

It is estimated that in both France and the UK, 5–10% of appointments with GPs are not kept.^1,2^ In the UK, GPs are paid via capitation, receiving a fixed payment per patient. Missed appointments mean lost medical time but have no financial impact on GPs. For them, missed appointments may be perceived positively (allowing the accomplishing administrative tasks, or the catching up on backlog), thus encouraging them not to worry about this phenomenon. Contrarily, in systems such as France’s, where doctors are paid mainly per service, missed appointments affect physician reimbursement.

The French healthcare system operates on the principle of universal coverage, where access to health care is considered a fundamental right. It is based on compulsory health insurance, primarily funded through social contributions and taxes. This compulsory health insurance covers two-thirds of basic healthcare expenses. For the remaining third, many French citizens also subscribe to complementary health insurance, often provided by private insurers. Those with annual incomes below 13,120 Euros (approximately 11 050 GBP) for a single person, can benefit from the State Health Coverage (SHC). This is a community-supported supplementary insurance aimed at ensuring that all citizens, regardless of their financial situation, can benefit from comprehensive and equitable healthcare coverage.

A French epidemiological study, ascribed 60% of missed appointments to patients who were beneficiaries of the French SHC.^1^ Several doctors' unions have raised concerns about missed appointments, citing them as disruptive to the organisation of health care. They have decried a *'consumerism of care'*,^3^ with some proposing compensation for missed consultations.^4^

Despite the enormity of this issue, there has been no comprehensive study in France that has thoroughly examined the underlying factors contributing to patient absenteeism from medical appointments. A retrospective study conducted in Scotland, involving a cohort of 824 374 patients tracked over a 3-year period, brought attention disorders, psychiatric conditions, social anxiety, and diminished cognitive capacity to the forefront as the primary drivers behind missed appointments.^5^ This study revealed a statistically significant increase in mortality rates among patients who missed more than two medical appointments within a year, leading to the conclusion that *'missed appointments represent a significant marker of short-term all-cause mortality risk, particularly among patients with mental pathology*'.^5^ Other studies have emphasised that missed appointments are more prevalent among young and economically vulnerable patients.^6–8^ These findings make us pose the question: should missed appointments be considered by health professionals as a warning sign identifying patients’ vulnerability, rather than just an instance of healthcare system misuse? Therefore, it is imperative to comprehend the underlying causes of missed appointments to enhance the quality of health care and mitigate health-related social disparities.

The objective of our pilot study was to investigate the factors contributing to patient non-attendance within a specific outpatient clinic.

## Method

We conducted a mixed-method study to explore the factors surrounding missed medical appointments, through qualitative telephone interviews and quantitative data collection from medical records.

For the practicality of this pilot study, the research was conducted exclusively at a single urban health centre. This health centre had 10 practising physicians, comprising seven regular physicians and three interns in ambulatory primary care operating under supervised autonomy (APCSA). Patients had multiple options for scheduling their medical appointments, including online booking, telephone reservation, or in-person appointment scheduling with the office secretaries. Appointments were automatically confirmed via short message service (SMS) or email, both at the scheduled time and the day before the appointment. Patients could cancel their appointment via a provided link in the SMS or email, or through a phone call.

All patients from this health centre who had missed at least one appointment during a fixed 15-day period were contacted for a qualitative telephone interview. We designed a semi-structured interview guide, which was pre-tested with non-medical individuals. These interviews were conducted by the principal investigator, FG, who had no prior familiarity with the patients at the urban health centre and had not previously worked there. Between 11 October 2021 and 23 October 2021, we reached out to all adult patients who 1) had missed appointments with any of the 10 GPs at the urban health centre, and 2) were proficient in either French or English. Patients were contacted within 48 hours of their missed appointment. In cases of non-response, a voicemail was left, with two additional attempts made to reach the patient within a week.

The investigator inquired about the context and reasons for their absence, and gathered social information to calculate a validated 'precariousness score', known as the EPICES (Evaluation of Deprivation and Inequalities in Health Examination Centres) score.^9^ This score ranges from 0 (indicating no precariousness) to 100 (representing maximum precariousness), with a threshold for precariousness set at 30.^9^

The qualitative interviews were transcribed verbatim, and a thematic analysis was conducted using a phenomenological approach. To ensure consistency in themes and codes, the first three interviews were joint-coded by LC, an attended physician of the urban health centre and an experienced qualitative researcher.

Simultaneously, quantitative data were extracted from the medical records of patients who had missed appointments. These data included demographic information (such as sex and age), the presence of SHC, the existence of chronic illnesses, the number of consultations throughout the year, and any experience of domestic violence noted.

During the qualitative interview, the investigator provided patients the opportunity to reschedule appointments with their preferred doctor. FG and LC subsequently assessed whether these rescheduled appointments were attended and, if so, determined their importance based on the outcome. Importance was categorised as follows: 'capital appointment' if missing the consultation could have significantly harmed the patient’s health (ranging from permanent disability to death), 'important' if the appointment involved a change in the usual treatment or an emergency visit with a specialist, and 'usual' if the appointment did not substantially alter the patient’s management.

No personally identifiable data were collected. During the phone call, participants provided oral consent to participate in the research and to the recording of the interview.

## Results

### Overview

**Figure 1.**
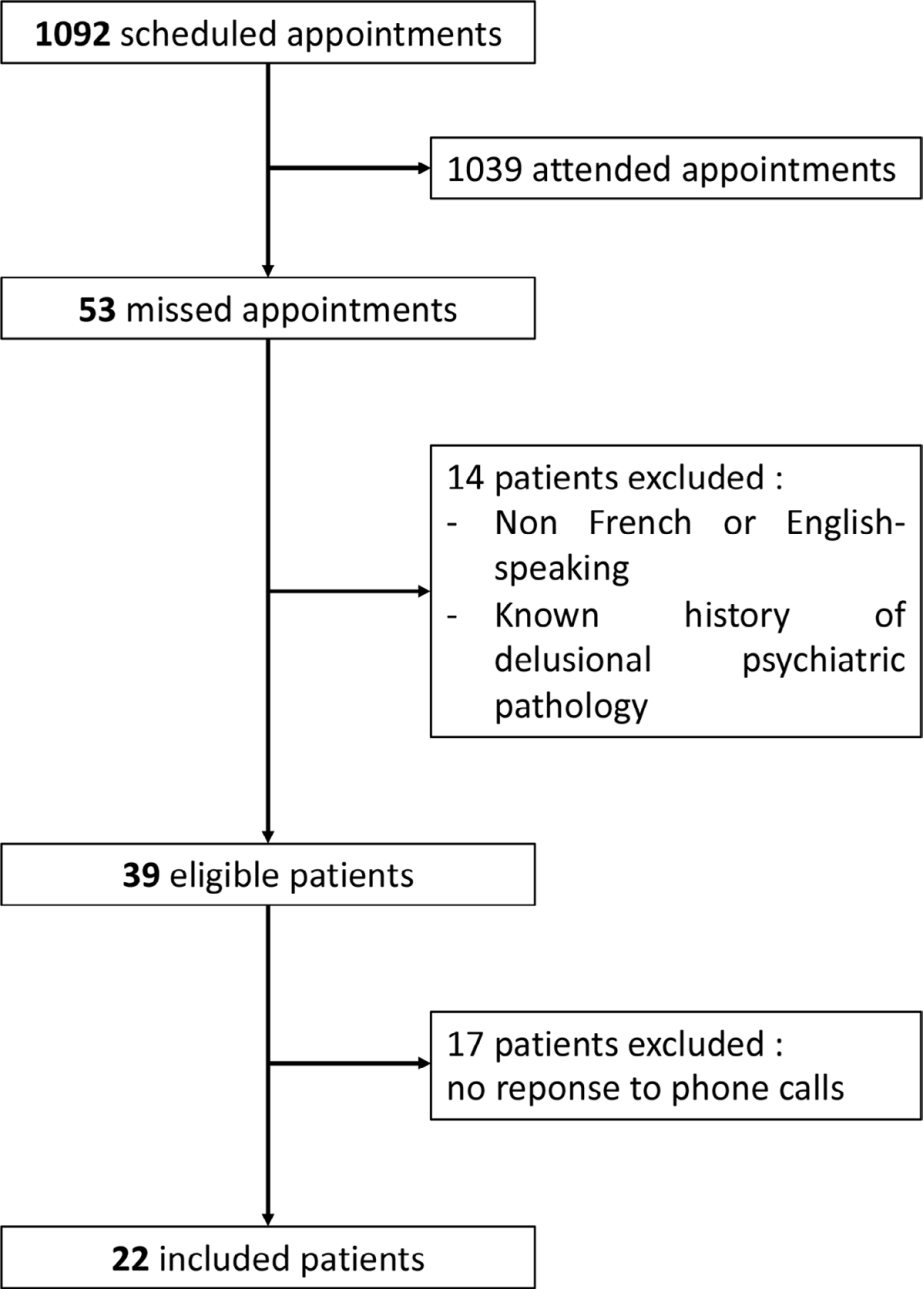
Flowchart summarising the inclusion of patients in the study

Out of the 1092 scheduled appointments over the study period, 53 (4.9%) were missed (Figure 1). Among the 580 appointments scheduled with attending physicians, 21 (3.6%) were missed, whereas 13 out of 328 appointments with locum tenens physicians were unattended (4.0%). Furthermore, out of the 184 appointments scheduled with APCSA interns, 19 (10,3%) were missed.

Among the 39 eligible patients of the study, 25 (64.1%) were recipients of SHC, and 14 (35.9%) were not.

Out of the 39 patients contacted, 22 (56.4%) responded. None of the patients declined to participate in the interview. Their characteristics are presented in Table 1. On average, the interviews lasted 10 minutes and 12 seconds. Data collection from medical records revealed that eight patients out of the included 22 (36.4%) were known to have experienced domestic violence.

**Table 1.** Summary of characteristics of patients who responded to the telephone interview

	Patients who participated in the interview (*n* = 22)	Primary health insurance office data for all patients of the urban health center (*n* = 6118)	*P* value
Sex F, n (%)	15 (68.2)	3302 (54.0)	0.49
Average age (sem)	40.3 (3.1)	33	
Min	19		
Max	76		
Age distribution			
Age 18–39, *n* (%)	11 (50.0)	2080 (34.0)	
Age 40–64, *n* (%)	10 (45.5)	2674 (43.7)	
Age ≥65, *n* (%)	1 (4.5)	844 (13.8)	
SHC, *n* (%)	15 (68.2)	3010 (49.2)	0.38
Chronic illness, *n* (%)	9 (40.9)	1346 (22.0)	0.15
of which chronic psychiatric illness, *n* (%)	5 (22.7)		
Experience of violence in patient history, *n* (%)	8 (36.4)		
History of illicit substance use, *n* (%)	6 (27.3)		
Born in a foreign country, *n* (%)	5 (22.7)		
Average EPICES score (sem)	41.3 (1.46)	47.7	
Min	8.3	0	
Max	88.2	100	
Number of consultations per year per patient			
Average	14		
>10 consultations in a year, *n* (%)	13 (59.1)	655 (10.7)	<0.05*
6–10 consultations in a year, *n* (%)	4 (18.2)	1333 (21.8)	0.99
2–5 consultations in a year, *n* (%)	1 (4.5)	3015 (49.3)	<0.05*
1 consultation in the year, *n* (%)	4 (18.2)	1107 (18.1)	0.98

Sem = standard error of the mean. SHC = state health coverage. Bold and asterisked = statistically significant.

For the patients benefiting from SHC, the factors contributing to their absence from medical appointments were varied and complex. Many of these factors were intrinsically linked to precarious circumstances, such as unstable employment and the fear of job loss, shifting work schedules, challenges in arranging childcare for single-parent households, and the demanding nature of medical and social follow-up involving multiple appointments.

Patients not covered by SHC cited more limited reasons for their missed appointments. These reasons also included work-related difficulties, simply forgetting the appointment, or resolving the health issue without remembering to cancel the appointment.

None of the patients mentioned absences related to issues with accessing care at the urban health centre or any concerns with the doctor-patient relationship.

The urban health centre employed an SMS-based appointment reminder system. However, several patients did not receive these reminders either because they lacked a phone or had changed their phone number. Some patients received the text messages but were unable to cancel appointments because their absence was sudden and unanticipated, resulting from unforeseen events occurring at the last minute.

### Theme 1: Work is health?

Frequently cited as the primary cause of missed appointments, prioritising work played an important role in patient absences:

*'It was always work* [the priority]*.'* (P3)

*'I put work before health.*' (P1)

Variable work hours and the demands of employment led to missed appointments:

*'As I was working, that day I was asked to work extra hours and I completely forgot about the appointment.'* (P2)

In addition, the fear of job loss loomed large, as illustrated by P21:

*'I found a new job, so I had to stay later in the morning so I couldn't come. I couldn't say no because I just started and if I have to go to the doctor at least every month, then the boss will think there’s something wrong.'* (P21)

Conversely, some patients noted that work had a positive impact on their morale, subsequently benefiting their health. P2 shared the following comment:

*'If I don't work, then my morale doesn't go so well, so there you go, my health follows.*' (P2)

### Theme 2: Complex social and family dynamics take precedence over care

Interviews revealed a landscape of social vulnerability, leading to challenging personal life management and numerous social appointments aimed at enhancing or maintaining precarious daily lives:

*'I had an appointment with the family allowances fund and the primary health insurance office. This month I paid my rent in three instalments, because I got the benefits in three instalments. That way I can manage. When I need to, I prostitute myself.* [...] *Yes, but it doesn't happen often and it’s been a long time.'* (P18)

Health care often took a backseat to pressing family and social concerns. Among the 10 women with SHC coverage in our study, six were single mothers, whose commitments 'never stop', with the obligation to ensure the follow-up of the whole family on the medical and social level, with several stakeholders:

*'The fact of accumulating my two jobs, because we live in an area where life is a bit complicated ...* [...] *Afterwards, you see, I never stop running, because you see, I have two children, and so I’m obliged to go and pick them up from school, to bring them back ...* [...] *so I can't rest in fact, we can't.'* (P3)

*'Yes, several times, social worker, children’s educator, educational assistant* [...] *in addition, after the death* [of her husband] *my children needed psychological follow-up.'* (P3)

### Theme 3: Absences linked to emotional and cognitive factors

Several interviews highlighted patients’ moral distress:

*'It’s tougher right now, since my daughter is no longer here. But then, I have to think about myself, I have two little boys too, so I'm holding on to them now. I don't know how long my daughter has been ... prostituting herself.* [...] *And all that, it’s pretty hard to take, I guess.*' (P10)

Another patient, aged >70 years, had mixed up the appointment date:

*'I've turned 20 multiple times, so, you know, at my age, when you have gone through five operations, 3 caesarean sections, and then you have muscle fatigue, you have been sick to the bone and you have continued to work...* [...] *it was a Monday, and I thought it was Wednesday.* [laughs] *And so I came, and they told me it was Monday. So that meant that they took me on Wednesday anyway.*' (P19)

### Theme 4: Absences linked to an appointment with a locum tenens or APCSA intern

Some patients reported skipping appointments owing to anxiety about seeing an unfamiliar doctor. This anxiety stemmed from the need to revisit and recount their medical history.

*'... because she was the substitute for my regular doctor. And it’s true that I already struggle with people I don't know, so I preferred not to come* [...] *Yes well, I thought about coming, but in fact I was too anxious and I decided not to.'* (P16)

### Evaluation of rescheduled appointments

Sixteen of the 22 patients accepted the rescheduled appointment proposed by the investigator during the interview. All these rescheduled appointments were attended and took place within 15 days of the interview.

The importance of these appointments was categorised into three levels: 'capital', 'important', and 'regular'. Two out of 16 patients (12.5%) had appointments deemed 'capital' for their health (for example, untreated displaced wrist fracture following an incidence of domestic violence, emergency hospitalisation for suicidal risk). Six patients (37.5%) had 'important' appointments (such as adjustments to background treatment or completion of official paperwork), while eight patients (50.0%) had 'regular' appointments.

## Discussion

### Summary

Patients who missed appointments were largely in vulnerable situations, as indicated by a high rate of SHC and a prevalence of chronic illnesses. Moreover, 36.4% of them had experienced violence in their lifetime. The systematic use of the EPICES score allowed us to estimate the level of vulnerability for each patient, unveiling extreme isolation or material hardships in their most fundamental life needs, often concealed from their healthcare providers.

Our study illuminated socioeconomic insecurity as a core concern. Complex life circumstances and interconnected causes were frequently cited to provide reasons for missing appointments; for example, work overshadowing health, demanding medical and social schedules leading to mental strain, family issues dictating daily routines, and psychological struggles. Contrary to common beliefs among healthcare professionals, mere forgetfulness was not the leading cause.^1,10^

### Strengths and limitations

The small sample size of our study does not allow us to draw formal conclusions. However, the design of this study is innovative. The reasons for missed appointments that we found are identical to those of larger-scale studies,^11–16^ suggesting methodological strength.

Our study achieved a response rate of 56.4%, surpassing the typical range of 30–50% for response rates in qualitative telephone surveys.^17,18^

The main limitations of our study include its single-centre design and the non-representative patient selection owing to the urban health centre’s location in a 'precarious' part of the city. Additionally, the study carries inherent limitations related to qualitative methodology, particularly recall bias and social desirability bias.

### Comparison with existing literature

The World Health Organization’s (WHO) model for social inequalities in health highlights that health care is only one element within the health spectrum.^19^ Care and health were clearly differentiated by some patients. Work, for instance, was described as a health factor often prioritised over care. Denantes *et al*, said: *'Patients in precarious socioeconomic situations live day-to-day and struggle to project themselves into the future.* […] *Some patients are unable to anticipate and combine their multiple organisational constraints*'.^20^ The work of sociologist Castel further supported this notion, stating: *'Precariousness is also a relationship to time. To control the future, a certain stability of the present is necessary. The rights constituting social property allow to plan one’s life. If we are deprived of them, we are obsessed with the present without knowing what tomorrow will bring. The rise of social insecurity is also a return to the "day-to-day" life'.* ^21^ There is a mismatch between the temporality of the individual, in the urgency of living, and the temporality of aid and health actions, requiring a projection into the future.^22,23^ Aligning care organisation with patients' temporal realities could mitigate appointment absences. Some proven interventions seem easily achievable; for example; appointment reminders via SMS or phone, the option to cancel appointments online, and potentially reducing the wait time by offering short-term appointments (within 24 or 48 hours).^2^

Another notable finding from our study was that 36.4% of patients who missed appointments had experienced violence, particularly domestic abuse. This data is consistent with the fact that an increase in demand for primary care appointments and missed medical appointments are known warning signs for identifying patients experiencing domestic violence.^24^ However, it would be interesting to see in a larger-scale study if this data is confirmed, which would indirectly show if GPs are asking patients about experiences of violence and how this information is recorded in the patient file.

### Implications for research and practice

Our study is single-centre, and our sample size is too small to draw formal and generalisable conclusions. Nevertheless, our data raise numerous questions regarding the organisation of the healthcare system, and provide avenues for improvement towards a more equitable system. This encourages us to continue this pilot study by proposing a national study based on a similar mixed methodology, for which we are currently seeking funding. For this extensive study, we aim to assess the relationship between past experiences of violence and missed medical appointments on a large scale. Additionally, we'll examine the potential statistically significant correlation between socioeconomic deprivation and missed medical appointments. Furthermore, we are contemplating an intervention-based methodology to test solutions aimed at reducing missed appointments and their associated health impacts.

Missed medical appointments could be viewed as a marker of vulnerability and recalling patients could make the healthcare system more equitable. All rescheduled appointments during our study were attended. Half of the patients who resumed an appointment had a noticeable interest in being seen again quickly in consultation. Using missed appointments as triggers for follow-up calls to absent patients, inquiring about their health and suggesting new appointments, presents a promising avenue to enhance care and mitigate health inequalities. Of course, this would entail additional time, often unpaid, for already busy healthcare professionals. Political awareness and support for this approach are paramount in order for it to be feasible.

As a result of this study, interventions have been implemented at the urban health centre. Patients who miss appointments now receive SMS reminders saying, '*You have missed your medical appointment. We hope you are well. Do not hesitate to reschedule*'. In addition, a mechanism to identify vulnerable patients has been established, with receptionists and medical secretaries reaching out to these vulnerable patients for health inquiries and rescheduling in case of missed appointments.

## Data

The dataset relied on in this article is available from the corresponding author on reasonable request.
